# Young’s Modulus Variation of the Deformable Cement Adhesives Under Thermal Action in LRHS

**DOI:** 10.3390/ma18235341

**Published:** 2025-11-27

**Authors:** Jacek Karpiesiuk, Tadeusz Chyzy

**Affiliations:** 1 Faculty of Civil and Environmental Engineering, Department of Civil Engineering, Warsaw University of Life Sciences, Nowoursynowska 166, 02-787 Warszawa, Poland; 2 Faculty of Civil Engineering and Environmental Sciences, Bialystok University of Technology, Wiejska 45A, 15-351 Bialystok, Poland; t.chyzy@pb.edu.pl

**Keywords:** lightweight radiant heating system (LRHS), Young’s modulus, deformation, compressive strength, thermal action, adhesives C2S1 and C2S2

## Abstract

Young’s modulus (*E*), one of the many material properties, changes in response to thermal actions. The magnitude of these changes also depends on the material used. This is particularly important when the materials used are components of lightweight radiant heating systems (LRHSs) without screeds. Adhesives or adhesive composites take over the role of the screed in LRHSs. The adhesives, which directly connect the thermal insulation layer and the floor, are responsible for the proper functioning of the heated floor. Therefore, changes in their Young’s modulus cause a loss of layer integrity and ultimately delamination of the floor. Thus, research was conducted on the variation of the Young’s modulus of deformable cement adhesive mortars, specifically types C2S1 and C2S2, used in LRHSs under thermal actions. The deformation values of adhesive mortar samples were measured in a thermal chamber, subjected to compressive strength tests, at temperatures from 30 °C to 50 °C. Deformation measurements of heated samples were performed using the extensometer technique. The measurement results were subjected to mathematical analysis using polynomial regression based on the least squares method and the “Madrid parabola” formulas. After analysis, it was assumed that the Young’s modulus *E* for the deformable C2S1 cement adhesive, depending on the thermal action taken in the study, falls within the range of 4600 MPa to 5800 MPa when the temperature is varied from 30 °C to 50 °C. Simultaneously, the Young’s modulus *E* remains constant over these temperatures, at 2300 MPa for the C2S2 adhesive. Knowledge of the Young’s modulus and other strength parameters of adhesive mortars connecting layers of lightweight heated floors or other partitions, subjected to temperature can directly impact their durability. This data can be used to analyse the performance of LRHSs and numerical calculation techniques for various building partitions, such as stairs, balconies, and terraces.

## 1. Introduction

**Figure 1:**
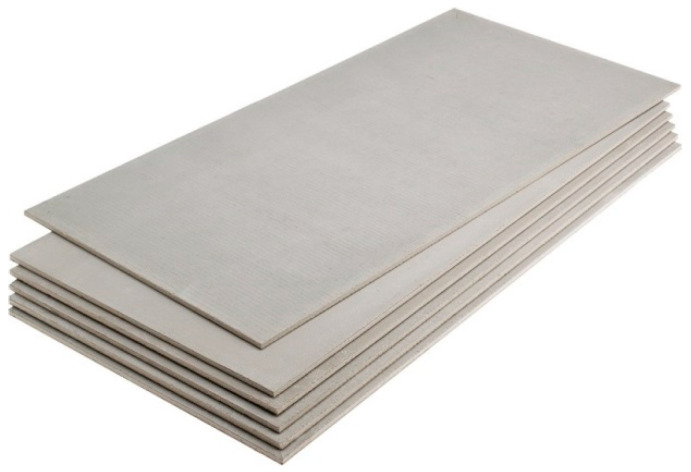
Construction board based on XPS thermal insulation (photo from Elektra Kardo).

In lightweight, layered floors, the subfloor is not a concrete screed, but rather various types of insulation boards, with varying degrees of thermal insulation. These boards are laid loosely or attached to the structural substrate with adhesive mortar, mechanical fasteners, or both. Depending on the type of flooring (ceramic, wood, wood-like, stone, various types of PVC, etc.), the flooring can be attached to the thermal insulation layer using manufacturer-specific adhesives. There is no standardised reference for laying ceramic tiles or other flooring horizontally directly on the thermal insulation layer using adhesive or a composite mesh with adhesive mortar. Examples of this floor construction include building boards, as in Figure 1, with an adhesive-mesh facing stiffening the insulation board.

Standard [1] identifies three basic adhesives used for bonding ceramic tiles: cementitious (C), dispersion (D), and reactive resin (R). The requirements of standard [1] are limited to selected strength tests, such as adhesion, shear, and specific material properties, such as transverse deformation. These tests are sufficient to introduce adhesives for sale and installation on concrete or other rigid substrates, such as OSB boards. However, these adhesive tests are insufficient for direct use on flexible substrates, such as various types of thermal insulation. Cementitious adhesive mortar is commonly used in construction for bonding ceramic and stone tiles, primarily due to its widespread availability and low price compared to other adhesives for this application. However, it is not always a good technical solution. In a lightweight radiant heating system (LRHS), thermal insulation is often covered with sheet metal or metal foil. Attaching the floor to such a surface using deformable cement adhesive mortars requires numerous additional procedures, such as impregnation, passivation, and others, which extend the final floor installation time and increase its costs. Another solution is the use of reactive resin-based adhesives. This group includes epoxy and polyurethane adhesives. Due to their physical and mechanical properties, they can also bond structural elements.

Where no metal layers are present on the floor, a more straightforward and cost-effective solution is to use deformable cementitious adhesives, which are described in [1], such as C2S1 or C2S2, reinforced with a glass fibre mesh. This was demonstrated in article [2], where these adhesives were used. Table 3 of this article confirmed that the strength of each layer of the composite lightweight heated floor without an aluminium foil layer was not exceeded. The lightweight floor in article [2] was tested for compressive, tensile, shear, and peel strength, and consisted of the following top layers:

- ceramic tiles;
- C2S1 adhesive reinforced with a GFRP layer;
- XPS insulation board attached to the concrete substrate with C2S1 adhesive.

The strength parameters used for individual materials of the lightweight heated floor in this article, such as Young’s modulus of elasticity and Poisson’s ratio, were assumed to be at a laboratory temperature of 20 °C. The potential for higher temperature performance was not considered when calculating the adhesive mortar stresses. As was laboratory tested, as described in article [2], the lightweight heated floor system is exposed to a temperature 12 °C higher than the room temperature at the adhesive layer. This means that to obtain reliable results for the stresses of the adhesive mortar and other layers of the lightweight heated floor, the strength parameters of the materials must be calculated to account for thermal actions. Therefore, it was decided to determine the elasticity modulus of C2S1 and C2S2 cement adhesive mortars when exposed to temperature. Generally, but not always, as temperature increases, Young’s modulus decreases due to the weakening of the bonding strength between the atoms of a given material, as confirmed by articles [3,4,5,6]. Experimental studies in [3] examined the effect of cement mortars prepared with various insulating aggregates, sand mortar, and sand mortar with admixtures on elevated temperatures (from 20 °C to 250 °C) in a thermal chamber. The results show that the variability of the mechanical parameters of thermal mortars is mainly caused by temperature in the cement paste. Although thermally insulating mortars retain their insulating properties at high temperatures, they change their mechanical strength, particularly at temperatures of 100 °C and above. Mortar with elastic admixtures demonstrated reduced susceptibility to changes in mechanical parameters up to 100 °C, but at higher temperatures, Young’s modulus decreased by 29% to 55%. However, the dynamic shear modulus and Poisson’s ratio, similarly to Young’s modulus, decreased with increasing temperature.

Furthermore, it should be confirmed that the decrease in Young’s modulus at temperatures between 200 °C and 250 °C in almost all mortars is consistent with the results of Phan [7], who observed more brittle fractures at 200 °C than at lower temperatures. Therefore, the conclusion is that thermal impact increases the stresses in the mortar material, which results from physical and chemical changes in its microstructure [8], including water evaporation, causing an increase in the internal pore pressure [9]. This, in turn, accelerates the hydration of unhydrated cement and the occurrence of microcracks [10]. When investigating the effect of temperature on the compressive behaviour of concrete [4], based on experimental data and constitutive models performed according to ASTM C39/C39M–20 [11], concrete lost 10–20% of its original compressive strength when heated to 100 °C and 30–40% at 260 °C, as confirmed in Table 1.

**Figure 2:**
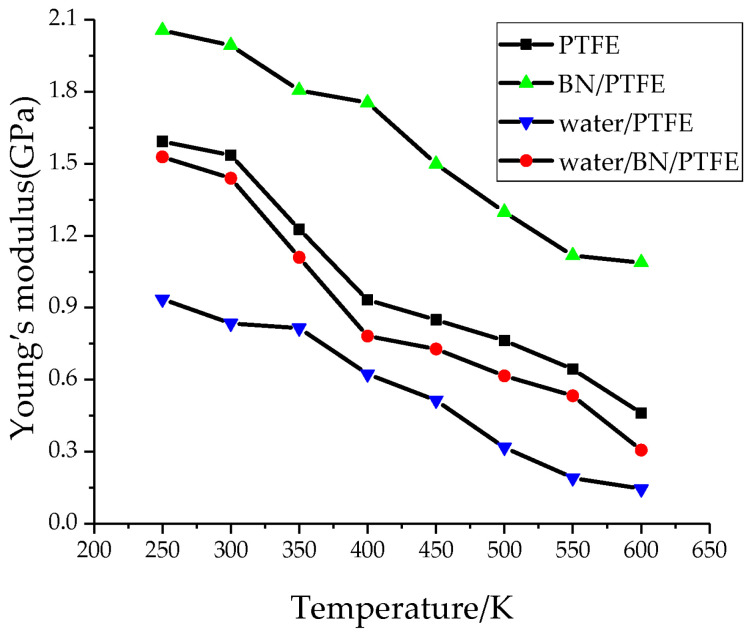
Young’s modulus versus temperature of the polytetrafluoroethylene PTFE, the boron nitride (BN) nanoparticles with PTFE, and PTFE materials under moisture. Reprinted from [13].

**Figure 3:**
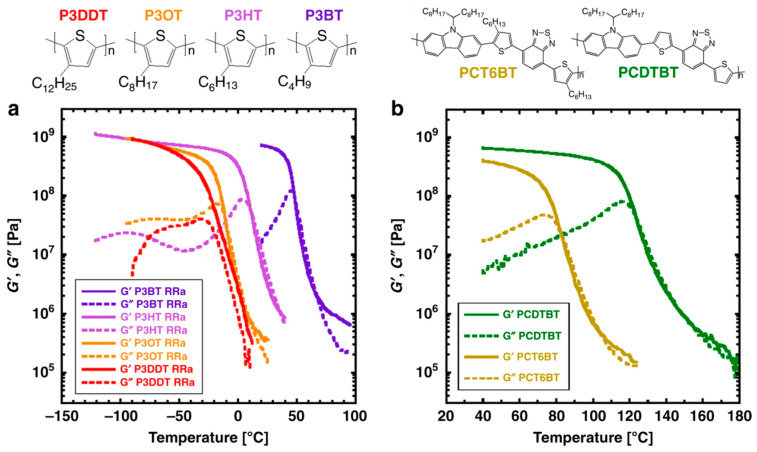
Locating the glass transition temperature using rheology. Reprinted from [14]. (**a**) Storage (G′) and loss (G″) moduli for regiorandom P3ATs with different side chain lengths (P3BT, P3HT, P3OT, and P3DDT); (**b**) G′ and G″ for PCDTBT and PCT6BT. Strain amplitude of 0.001, frequency of 1.0 rad/s, heating rate of 5 °C/min. The glass transition temperature is taken at the peak in G″.

This was noted in the stress–strain curves of concrete and was consistent with the damage changes observed in [12] under thermal exposure. A similar phenomenon was observed in article [5] concerning evaluating the strength and modulus of elasticity of polymer-modified cement concrete under thermal exposure in the operating temperature range up to 60 °C. Article [5] concluded that: “The application of different service temperatures leads to significant differences in the mechanical parameters of the mortars investigated, so that temperature must be considered as a significant factor in modelling of the material behaviour”). In article [6], which examines, among other things, the effect of temperature on the values of the elastic modulus and yield strength of polymers and polymer nanocomposites, which are also components of deformable cement mortars, a process of decreasing Young’s modulus with increasing temperature is observed. This is confirmed by Figure 2 and Figure 3, which concern the longitudinal (Young’s modulus, E) and transverse (shear modulus, G) elasticity of the material, respectively, as referenced in articles [13,14]. Both Young’s modulus (E) and shear modulus (G) typically decrease with increasing temperature; however, their rates of decrease differ due to material-specific behaviour and temperature-dependent changes in Poisson’s ratio.

The literature cited above [2,3,4,5,6,7,8,9,10,11,12,13,14] confirms that cement mortars and cement concretes with polymer additives are similar in terms of the primary material used—cement and aggregate constituting over 90% (Table 3 of [15] and Table 4 of [16]) to deformable cement adhesives of the C2S1 and C2S2 types. Hence, these deformable cement adhesives may also be characterised by similar stress–strain relationships, and Young’s modulus should decrease, similarly to cement mortars or concretes with polymer additives. It is only necessary to verify their values and how much they will decrease depending on the thermal impact. It is also important to assume the magnitude of the thermal action on Young’s modulus occurring in the LFHS system under consideration. Article [17] and standard [18] confirm that the maximum supply temperature in an LFHS system should not exceed 50 °C. Therefore, there is no need to perform tests on the modulus of elasticity in LFHS at temperatures higher than 50 °C. The authors of articles [17,18] conducted experimental studies on the supply temperature of lightweight-heated floors. They concluded that the typical supply range, comfortable for humans, includes low temperatures, usually not exceeding approximately 50 °C. In practical applications, especially when a heat pump powers the system, the preferred values are 35–45 °C. This allows for maintaining comfortable user conditions on the floor surface and not exceeding the maximum temperatures at which the floor, especially wood or wood-based materials, can be used. The authors of [17,18] emphasise that due to the properties of wooden floors regarding humidity and temperature, the maximum design temperature on the floor surface should be 27–29 °C. In such cases, the supply temperatures on the coil should not exceed 35–45 °C. Therefore, the maximum design supply temperature for light-heated floor systems without screeds should not exceed 50 °C, especially when the floor is made of wood-based materials. These design assumptions are confirmed in standard [18] and article [19]. Moreover, as stated in the above literature [3,4,5,6,7,8,9,10,11,12,13,14], the variability of strength parameters, including Young’s modulus of deformable C2S1 and C2S2 adhesive mortars, depends on their internal composition, particularly the amount of water and polymers used to make the adhesive mortars elastic.

## 2. Materials and Methods

Laboratory experimental studies were conducted to determine the Young’s modulus of cement adhesive mortars at various exposure temperatures. The following adhesive mortars were used for this purpose:

- Sika Ceram C2S1 deformable cement adhesive;
- Shonox C2S2 deformable cement adhesive.

The main components of these adhesives are cement, most often Class I, and aggregate in the form of quartz sand, which constitutes over 90% of the total mass [20], leading to their classification as cementitious adhesive mortars. Additives such as methylcellulose, dispersion powder, fibres, hydrophobic additives, and setting and hardening accelerators complement the composition. Each of these ingredients serves a specific function. Redispersible polymer powder (RDP) enhances adhesion and deformability. Hydroxypropylcellulose (HPMC) or other cellulose ethers modify the adhesive’s consistency and open time. Calcium formate accelerates the hydraulic setting and improves adhesion, while polymer fibres enhance deformability and crack resistance. The mentioned additives improve the performance properties of adhesive mortars, as confirmed in [16,21], and their quantity determines the strength and other characteristics described in the standard [1]. This standard categorises cementitious adhesives into two classes: Class C1, indicating normal adhesion to a flat, rigid substrate of ≥0.5 N/mm^2^, and Class C2, representing high adhesion of ≥1 N/mm^2^. The values given indicate the tensile strength perpendicular to the faces of the adhesive mortar when attached to the concrete slab substrate. The S1 and S2 designations indicate the lateral deformability of cementitious adhesives, where S1 denotes adhesives deformable within the range of 2.5–5 mm, and S2 indicates high deformability >5 mm [22]. It should be noted that not every adhesive classified as C2 also meets the requirements of class S1 or S2. Better adhesion and elasticity can be achieved using different polymers. Polymers that provide high adhesion do not necessarily offer elasticity (and vice versa). These types of cementitious adhesive mortars, described in standard [1], can be used in a lightweight heated floor system (LRHS).

**Figure 4:**
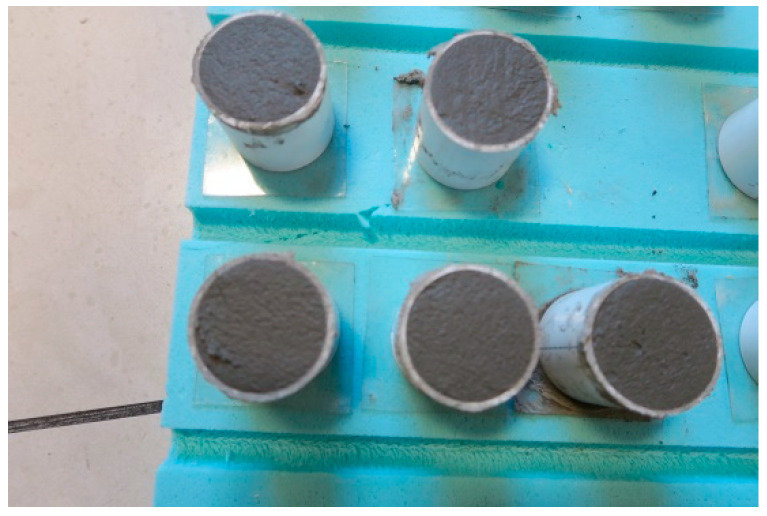
Five samples of Sika Ceram C2S1 adhesive mortar before demoulding, dimensions 50 mm × 50 mm. Reprinted from [23].

Five cylindrical samples of each type of C2S1 and C2S2 adhesive mortar were prepared, each measuring 50 mm in diameter and 50 mm in height. Examples of Sika Ceram C2S1 adhesive mortar samples before demoulding are shown in Figure 4. After 6 months of curing, they were milled. The need for milling the samples arose because, after removal from the moulds, the PVC samples exhibited minor irregularities (primarily horizontal at the base and top of the sample), as well as minor depressions and scratches. The pressing machine requires perfectly even surfaces to ensure that the initial pressing process does not distort the initial strength results. Therefore, before compressive strength testing, the cementitious adhesive samples were levelled on a lathe to achieve even surfaces. Samples of C2S2 adhesive mortar were prepared similarly. Ultimately, after mechanical machining, the test dimensions of all adhesive mortar samples were 46 mm (C2S1) and 46.6 mm (C2S2) in diameter, and all 48 mm high. After milling, the three best samples were selected for axial compressive strength testing. The base measurement of each adhesive mortar sample, on which the strain gauges were placed, was 37.5 mm.

**Figure 5:**
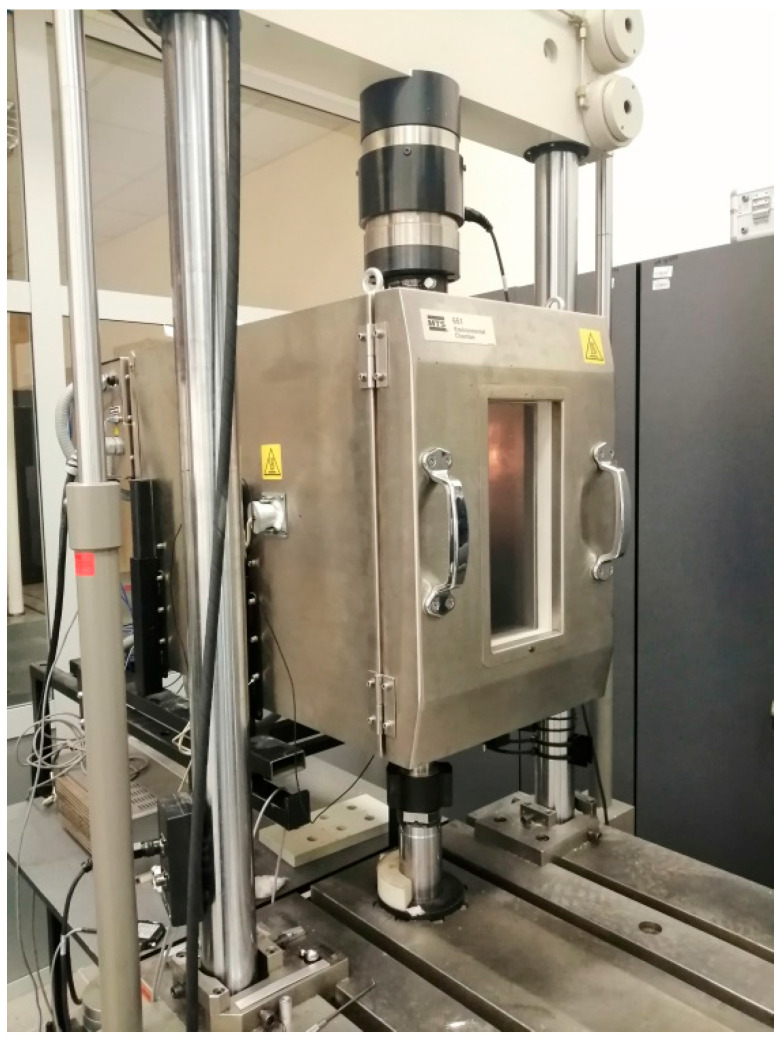
The MTS 651 thermal chamber for testing at temperatures up to 315 °C, working in conjunction with the Load Unit MTS 322 testing machine.

Three samples of each adhesive mortar were placed in an MTS 651 environmental chamber (MTS Systems, Eden Prairie, MN, USA) (Figure 5) and subjected to compression using an MTS 322 (MTS Systems, Eden Prairie, MN, USA) Load Unit hydraulic testing machine with a controlled clamping force and an axial load range of the first actuator of ±50 kN. The testing machine can control the testing process using displacement, force, or deformation using an MTS TestStar II 90.01 controller (MTS Systems, Eden Prairie, MN, USA).

**Figure 6:**
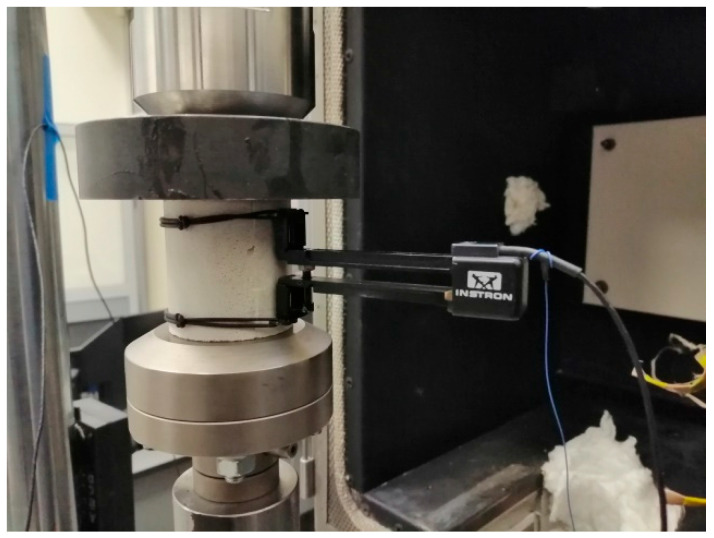
A sample of C2S1 adhesive mortar was placed in a compression machine with a load range of ±50 kN and a working stroke of ±75 mm, where an Instron 2620-601 extensometer was installed.

**Figure 7:**
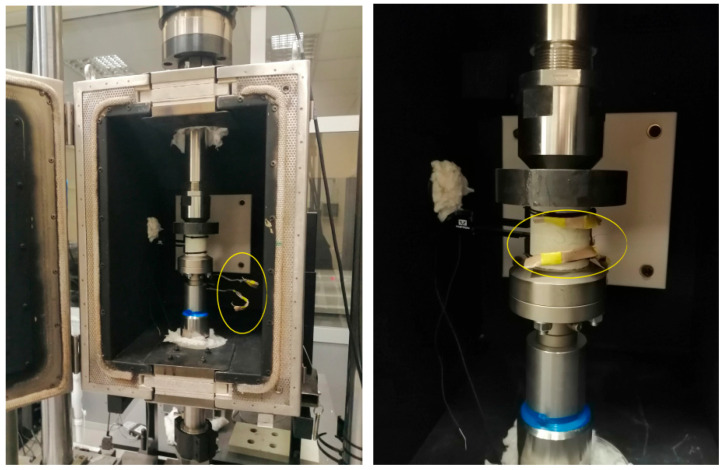
Temperature sensors for measuring temperatures in the range of 30–50 °C were attached to the test samples—marked in yellow.

An Instron 2620-601 extensometer (Instron, Norwood, MA, USA) with a measurement accuracy of 0.15% FSD was placed on each sample, allowing displacement measurements at varying temperatures ranging from 30 °C to 50 °C. This extensometer, fixed with rubber O-rings, was used to measure longitudinal deformations during the compressive strength test to determine Young’s modulus of the adhesive mortars. The method of attachment is shown in Figure 6. Temperature sensors were attached to each sample to directly measure their outer surface, as shown in Figure 7.

After placing the instrumentation on the samples, the compressive strength test was performed, and displacements were simultaneously measured. The initial temperature was 20 °C. The test speed for the deformable C2S1 and C2S2 adhesive mortars was V = 0.6 mm/min. The speeds were set at the same level as previously tested samples of the same adhesives under laboratory conditions at 20 °C without using a thermal chamber, as described in article [23]. Compression tests in a thermal chamber were conducted at the Bialystok University of Technology laboratory.

## 3. Results and Discussion

The study’s main objective was to determine the Young’s modulus *E* and the compressive strength of deformable C2S1 and C2S2 adhesive mortars. These data are necessary for numerical calculations and are not described in the standard [1]. After reaching the assumed temperature values, measurements were performed on three selected cylindrical samples described in Section 2. The test began at 20°C and ended at 50 °C with a modulus of 10 °C. Initial measurement readings began at 25 °C, but measurements starting from 30 °C were used for the computational analysis.

According to the American standard [24], initial readings should not be included in the calculations due to possible small shifts during the first small load increment when determining Young’s modulus. With this in mind, and using the calculation formula of Hooke’s law (1) for the stress–strain relationship, preliminary Young’s moduli of the tested adhesive mortars were determined experimentally.

(1) $$\sigma =\frac{F}{A}$$

σ—material stress;
*F*—compressive force;
*A*—surface area of the cylindrical sample, calculated as the area of a circle, equal to πr^2^.

**Figure 8:**
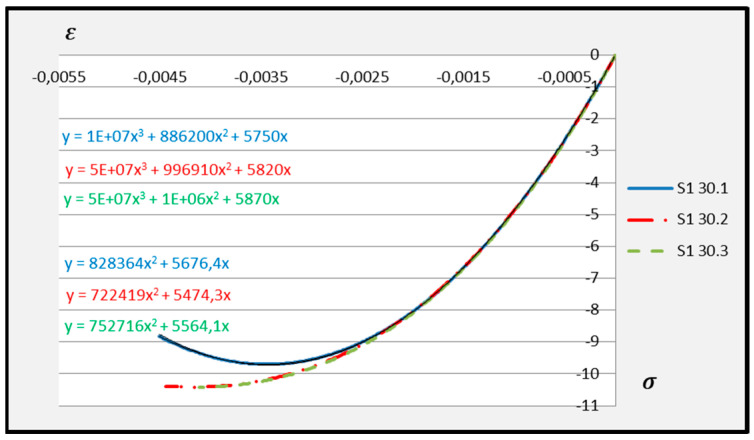
Dependence *σ* on *ε* C2S1 deformable adhesive for three samples under compression at 30 °C.

**Figure 9:**
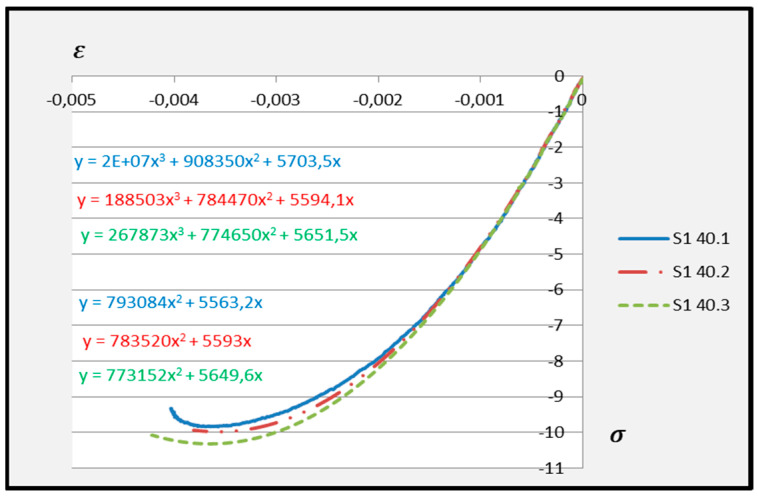
Dependence *σ* on *ε* C2S1 deformable adhesive for three samples under compression at 40 °C.

**Figure 10:**
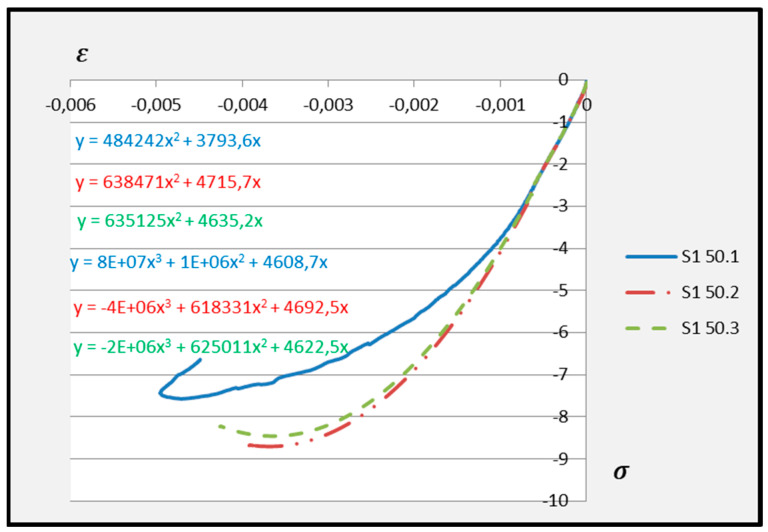
Dependence *σ* on *ε* C2S1 deformable adhesive for three samples under compression at 50 °C.

**Figure 11:**
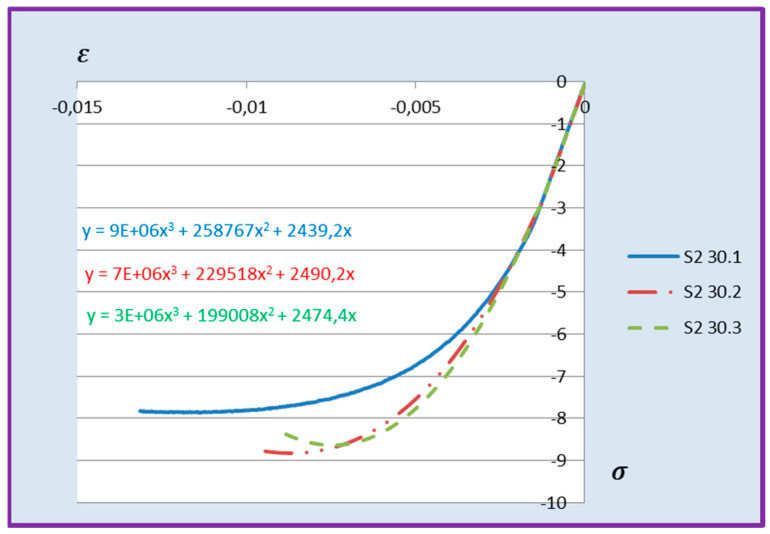
Dependence *σ* on *ε* C2S2 deformable adhesive for three samples under compression at 30 °C.

**Figure 12:**
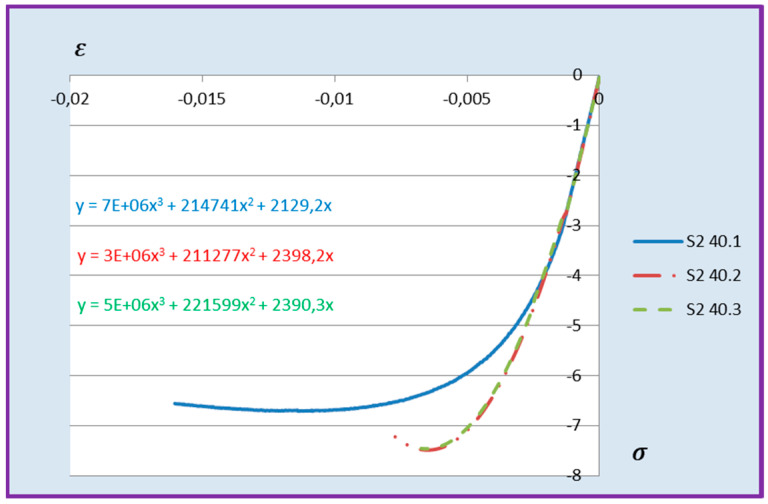
Dependence *σ* on *ε* C2S2 deformable adhesive for three samples under compression at 40 °C.

**Figure 13:**
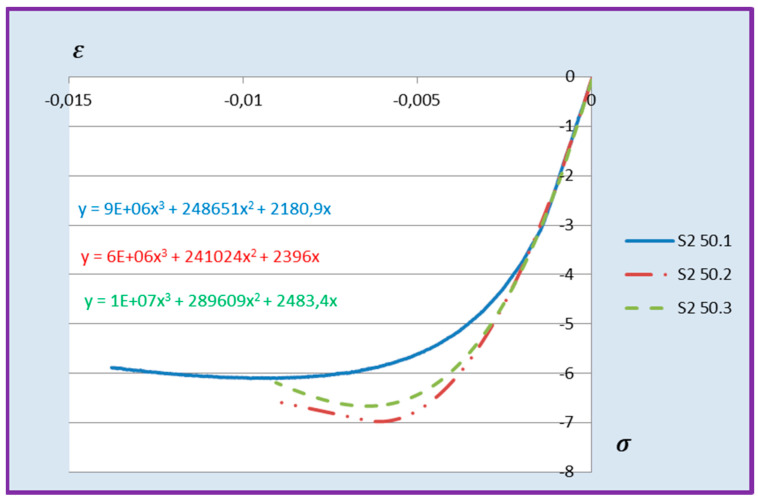
Dependence *σ* on *ε* C2S2 deformable adhesive for three samples under compression at 50 °C.

Based on the above formula, quadratic functions characterising the relationship between stress *σ* and longitudinal strain *ε_m_* during compression of cement adhesive mortars were determined. Next, trend lines of second- and third-degree polynomial functions were determined using the least squares method for the experimental data of the C2S1 adhesive mortar. In contrast, only trend lines of third-degree polynomial functions were chosen for the C2S2 adhesive mortar, as they better reflected the research data. The stress–strain relationships for each tested C2S1 and C2S2 adhesive mortar sample, along with the formulas of second- and third-degree polynomial functions, depending on thermal exposure in the range of 30–50 °C, are presented in Figure 8, Figure 9, Figure 10, Figure 11, Figure 12 and Figure 13.

The stress–strain relationship in the tested cement mortars subjected to compression is not linear. Its shape resembles the stress–strain relationship of a compressed wall. A detailed explanation of the stress–strain relationship for C2S1 and C2S2 cement adhesive mortars is presented in [23]. In the case of cement-based adhesive mortars, the initial compression phase of these samples is characterised by an increase in stresses with strain to a maximum value. Then, as strain increases, stresses decrease. For this reason, a parabolic graph of the cement adhesive’s third-degree function was used for calculations. As indicated in [23], during the initial compression phase of the adhesive, minimal disturbances occur (despite prior proper sample preparation) due to the initial adjustment of the loading force to the adhesive mortar. These disturbances subside very quickly, not exceeding the maximum stress level of approximately 1%. In such a situation, as recommended in [23], the vertical stress axis can be shifted to the point of intersection of the curve with the strain axis, which was applied. Thus, the approximation of the dependence of σ on ε at point 0 was made. This initial adjustment of the force to the test specimen introduces an error that manifests itself as an initial distortion of the stress–strain relationship. This error was greater when testing the C2S1 adhesive at 50 °C and the C2S2 adhesive at temperatures between 30 °C and 50 °C, as evidenced by the d coefficient values of the third-degree polynomial function not being equal to 0 but being approximately 10 times greater than the d coefficients of the C2S1 adhesive at 30 °C and 40 °C. Hence, Figure 8 and Figure 9 show a greater convergence of the function graphs compared to Figure 10, Figure 11, Figure 12 and Figure 13.

**Figure 14:**
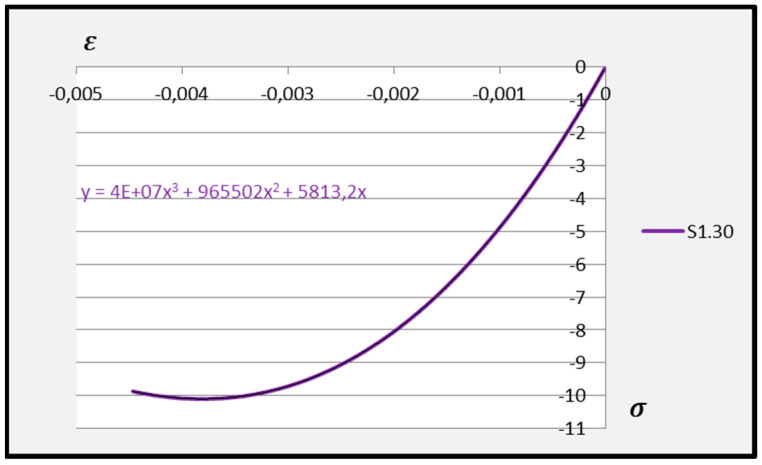
Averaged stress–strain dependence function for C2S1 deformable adhesive under compression at 30 °C.

**Figure 15:**
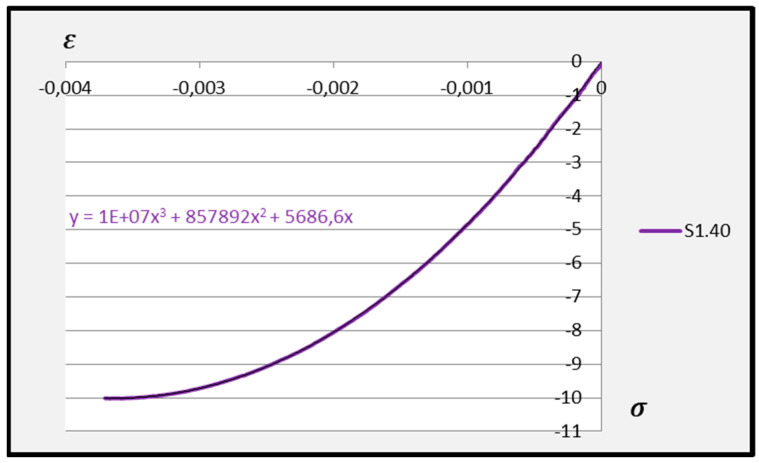
Averaged stress–strain dependence function for C2S1 deformable adhesive under compression at 40 °C.

**Figure 16:**
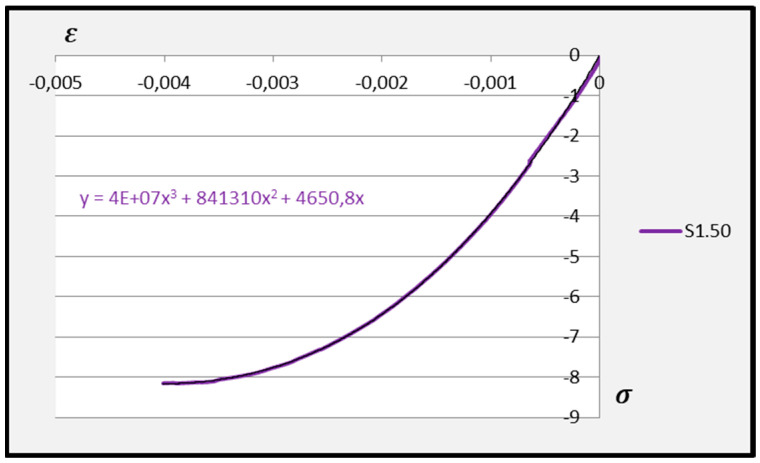
Averaged stress–strain dependence function for C2S1 deformable adhesive under compression at 50 °C.

Further analysis of the polynomial functions of the two deformable adhesives was based on third-degree polynomials to better represent the experimental results. Arithmetic means of the polynomial functions were then calculated based on the test results for the individual adhesives at 30 °C, 40 °C, and 50 °C. The averaged third-degree functions of the C2S1 adhesive mortars are shown in Figure 14, Figure 15 and Figure 16, while all averaged functions of the C2S1 and C2S2 mortars are recorded in Table 2.

The tangent to a third-degree polynomial can be determined based on these functions. This is accomplished by calculating the derivative of the polynomial, where the tangent at a given point x_0_ has the same slope as the function’s graph. This slope equals the value of the function’s derivative at that point. The averaged graphs of the third-degree functions C2S1 and C2S2, presented in Table 2, do not include the constant d, meaning the graphs originate at point 0, which is consistent with the standard [24], and the derivative of the third-degree function has the form of Formula (2).

y’(x) = 3ax^2^ + 2bx + c (2)

The same mathematical principle was used to determine the tangent of the second-degree polynomial, as described in the articles by Bednarski et al. [25] and Nejati et al. [26]. The rectilinear part of the second-degree polynomial of the stress–strain relationship was used to determine the value of the preliminary longitudinal stiffness modulus *E*. For this purpose, a tangent or secant line should be drawn at point x = 0 to the determined functions of C2S1 Sika Ceram and C2S2 Shonox mortars from Table 2. Calculations for the tangent equation to the function graph were performed using Formula (3).

y_1_ − y(x_0_) = y′(x_0_)·(x − x_0_) (3)

For S1 adhesive mortars, based on the functions presented in Table 2, depending on temperature, Young’s modulus *E* is determined as follows:

The S1.30 function has the following form: *y* = 4 × 10^7^x^3^ + 965,502x^2^ + 5813x.

Assuming *x*_0_ = 0, determine the derivative:

*y*′ = 120,000,000*x^2^* + 1,931,004x + 5813 and we calculate its value for *x*_0_ = 0:

*y′*(*x*_0_) = 5813, where *y*(*x*_0_) = 0 the tangent equation has the form:

*y_S_*_1.30_ − 0 = 5813·(*x* − 0), hence:

*y_S_*_1.30_ = 5813*x*

So, Young’s modulus is:

$${E_{S}}_{1.30}\;=\;\mathrm{tg}\text{𝛼}\;\;\frac{y_{S1.30}}{x}=5813\;\mathrm{MPa}\approx 5810\;\mathrm{MPa}.$$

The calculated Young’s modulus of adhesive mortar S1.30 when determining the tangent to the graph of the averaged third-degree polynomial function indicates that it is ultimately expressed as the numerical value at x of each function of this polynomial. Performing analogous calculations for each adhesive mortar S1 and S2 at temperatures of 30–50 °C, we obtain the following Young’s modulus values:

*E_S_*_1.30_ = 5810 MPa, *E_S_*_1.40_ = 5680 MPa, *E_S_*_1.50_ = 4650 MPa.

*E_S_*_2.30_ = 2470 MPa, *E_S_*_2.40_ = 2300 MPa, *E_S_*_2.50_ = 2350 MPa

These are average values that can be expressed approximately. According to the research described in article [27] on the effect of different aggregate batches, aggregate type, and water/cement ratio on the variation of the elastic modulus of concrete, it was shown that the elastic modulus should be treated probabilistically. It is best to assume a lower elastic modulus value relative to the average value, as it is safer for strength calculations. Therefore, Table 3 summarises the Young’s modulus of elasticity for deformable C2S1 and C2S2 adhesive mortars, depending on thermal actions, based on mathematical calculations using Formula (3) and the indications in article [27]. Additionally, the table includes data on the average compressive strength of C2S1 and C2S2 adhesives and the corresponding average strains and displacements of the tested adhesive mortars. It should be noted that compressive strength values decrease with increasing temperature, while deformation increases. The studies cited in the above articles [2,3,4,5,6,8,9,10,11,12] confirm this.

Describing the values of the approximately average Young’s modulus, we have:

*E_S_*_1.30_ = 5800 MPa, *E_S_*_1.40_ = 5600 MPa, *E_S_*_1.50_ = 4600 MPa

*E_S_*_2.30_ = 2400 MPa, *E_S_*_2.40_ = 2300 MPa, *E_S_*_2.50_ = 2300 MPa

The values of Young’s modulus given in Table 3 were supplemented with statistical data—measurement uncertainties of Young’s modulus, which are recorded in Table 4. The standard uncertainty of the mean u, and the expanded uncertainty with a coverage factor k = 2 (approximately 95% confidence), are denoted as *U* = 2*u*. The table provides the mean value and *U* (±). The ±U (k = 2) results are small compared to the values (e.g., ±70 MPa for ~5800 MPa) for C2S1 adhesives or ±30 MPa for C2S2 adhesive at 30 °C, which provides reasonable precision. The lower precision applies to C2S2 adhesives, as the ±U (k = 2) values, which are approximately ±180 MPa at 40 °C and 50 °C relative to approximately 2300 MPa, are approximately 8% of the value. Their deviation from the mean is less than 10%, which can be assumed to be correct.

The average values of Young’s modulus *E_S_*_1.30_, *E_S_*_1.40_*, E_S_*_1.50_, *E_S_*_2.30_, *E_S_*_2.40_, *E_S_*_2.50_, calculated by the least squares method in Excel based on the third-degree polynomial function from experimental data, were verified using the so-called “Madrid parabola” method. Analysis of the formulas given in Eurocode 2 [28] will indicate whether they are appropriate for calculating the Young’s modulus of elasticity of deformable adhesive mortars at elevated thermal action up to 50 °C based on the “Madrid parabola”. The formulas for the Madrid parabola (4) and (5) are given in Eurocode 2 [28] (point 3.1.7) in the following form:

(4) $$\sigma_{c}=f_{c}[1\;-\;(1\;-\;\;\frac{\varepsilon_{c}}{\varepsilon_{c2}}{)}^{n}],\mathrm{dla}0\leq \varepsilon_{c}\leq \varepsilon_{c2}$$

*σ_c_* = *f_c_*, dla *ε*_*c*2_ ≤ *ε_c_* ≤ *ε*_*cu*2_ (5)

where:

*f_c_*—computational strength of ordinary concrete, e.g., C12/15 ÷ C50/60, compressive strength;
*ε_c_*—current concrete deformation;
*σ_c_*—compressive stress in concrete corresponding to the *ε_c_*, deformation;
*ε_c_*_2_—the smallest deformation in concrete for which the stresses achieved are equal to the strength of concrete for compression (for normal concrete *ε_c_*_2_ = 0.002);
*ε_cu_*_2_—limiting deformation of concrete (for ordinary concrete *ε_cu_*_2_ = 0.0035);
*n*—exponent (for ordinary concrete equal to 2).

The arithmetic means of the C2S1 compressive strength from three samples in 30–50 °C—own names S1.30, S1.40, S1.50—are equal, respectively: *f_cS_*_1.30_ = 10.1 MPa, *f_cS_*_1.40_ = 10.0 MPa, *f_cS_*_1.50_ = 8.15 MPa, respectively, while the arithmetic means of the C2S2 compressive strength from three samples in 30–50 °C—own names S2.30, S2.40, S2.50—are equal, respectively: *f_cS_*_2.30_ = 8.44 MPa, *f_cS_*_2.40_ = 7.22 MPa, *f_cS_*_2.50_ = 6.58. Both these data were taken from the experimental research. The smallest deformations for these average strength values are *ε_c_*_2.*S*1.30_ = 0.00377, *ε_c_*_2.*S*1.40_ = 0.00381, *ε_c_*_2.*S*1.50_ = 0.00401 for type C2S1 and *ε_c_*_2.*S*2.30_ = 0.00933, *ε_c_*_2.*S*2.40_ = 0.00803, *ε_c_*_2.*S*2.50_ = 0.00726 for type C2S2.

Formula (4) from the standard [28] for a third-degree polynomial should be written as follows:

$$\sigma_{c}=f_{c}[1\;-\;(1\;-\;\;\frac{\varepsilon_{c}}{\varepsilon_{c2}}{)}^{3}]$$

It was noticed that the transformation of this formula allows it to be used to calculate the coefficients of the third-degree polynomial and thus to determine the value of the third degree polynomial of C2S1 and C2S2 adhesive mortars in mathematical terms, namely:

$$\sigma_{c}=f_{c}[1\;-\;(1\;-\;3\frac{\varepsilon_{c}}{\varepsilon_{c2}}+\;3\frac{\varepsilon_{c}^{2}}{\varepsilon_{c2}^{2}}-\;\frac{\varepsilon_{c}^{3}}{\varepsilon_{c2}^{3}})]$$

$$\sigma_{c}=f_{c}(3\frac{\varepsilon_{c}}{\varepsilon_{c2}}-3\frac{\varepsilon_{c}^{2}}{\varepsilon_{c2}^{2}}+\frac{\varepsilon_{c}^{3}}{\varepsilon_{c2}^{3}})$$

In simple terms, this can be written using Formula (6) as:

(6) $$\sigma =\sigma_{0}\;(3\frac{\varepsilon }{\varepsilon_{0}}-3\frac{\varepsilon^{2}}{\varepsilon_{0}^{2}}+\frac{\varepsilon^{3}}{\varepsilon_{0}^{3}})$$

where, analogously to the book [29], in the third-degree polynomial system we have:

*σ*_0_* = *$f_{c}$ (aniaxial compressive strength of adhesive), *ε*_0_ = 3*σ*_0_*/E*;
*E*—longitudinal elasticity modulus of adhesive;
$\varepsilon_{c2}\;$* = ε*_0_—the smallest deformation of the test material corresponding to its strength;
$\varepsilon_{c}$* = ε*—strain corresponding to stress σ.

After transforming Formula (6), we have Formula (7) reduced to a polynomial of the third degree:

(7) $$\sigma =\frac{\sigma_{0}}{\varepsilon_{0}^{3}}\varepsilon^{3}-\frac{3\sigma_{0}}{\varepsilon_{0}^{2}}\;\varepsilon^{2}+\frac{3\sigma_{0}}{\varepsilon_{0}}\;\varepsilon$$

After approximating the third-degree polynomial we have:

y = ax^3^ + bx^2^ + cx (Table 2); hence, for *ε* = x we have the following coefficient values:

$$a\;=\frac{\sigma_{0}}{\varepsilon_{0}^{3}};\;b\;=\;-\;\frac{3\sigma_{0}}{\varepsilon_{0}^{2}};\;c\;=\;\frac{3\sigma_{0}}{\varepsilon_{0}}$$

Substituting the average values of compressive strength $\sigma_{0}$ and strain $\varepsilon_{0}\;$from the experiment (Table 3), we obtain the following third-degree polynomial functions of each adhesive at a temperature of 30–50 °C:

S1.30—y = 188,493,910x^3^ – 2,131,866x^2^ + 8037x

S1.40—y = 180,811,093x^3^ – 2,066,671x^2^ + 7874x

S1.50—y = 126,393,427x^3^ – 1,520,513x^2^ + 6097x

S2.30—y = 10,391,961x^3^ – 290,871x^2^ + 2714x

S2.40—y = 13,944,102x^3^ – 335,913x^2^ + 2697x

S2.50—y = 17,195,548x^3^ – 374,519x^2^ + 2719x

After rearranging the equations with coefficients, we have:

$$\sigma_{0}=\;\frac{c\;}{3}\varepsilon_{0};\;\sigma_{0}=\;\frac{b\;}{3}\varepsilon_{0}^{2};\;\mathrm{hence}$$

$$\varepsilon_{0}=\frac{c}{b\;};\;\sigma_{0}=\frac{c^{2}\;}{3b}$$

Substituting $\varepsilon_{0}$ and $\sigma_{0}$ into the equation *ε*_0_ = 3*σ*_0_/*E* we have:

$$E\;=\;\frac{3\sigma_{0}}{\varepsilon_{0}}=c$$

This confirms that the value of Young’s modulus in a third-degree polynomial is the coefficient c at x.

However, the compressive strength values based on the transformed formula $\sigma_{0}$ = $\frac{c^{2}\;}{3b}$ of the “Madrid parabola” for individual adhesives and temperatures are consistent with the data in Table 3, confirming the correctness of the calculations and transformations performed. Simultaneously, it was noted that the mathematical calculations leading to the calculation of the Young’s modulus of deformable C2S1 and C2S2 adhesive mortars, based on the “Madrid parabola” formulas, are overestimated. This may be due to several reasons, including the fact that the “Madrid parabola” formulas were based on concrete and masonry mortars, not deformable adhesives with increased elasticity, as well as the possible effect of elevated temperatures up to 50 °C during testing of adhesives in a thermal chamber. The potentially significant impact of temperature on the increased Young’s modulus results during calculations based on the “Madrid parabola” formulas was confirmed in article [23]. There, the Young’s modulus of these deformable adhesives was tested only at 20 °C. In article [23] a comparison of the values of Young’s modulus and compressive strength of deformable adhesives, resulting from the experiment and the “Madrid parabola” formulas, was made. The results from the experiment and mathematical calculations were also overestimated, but slightly (more overestimated for the adhesive with higher deformability, i.e., C2S2), as presented in Table 5. Table 6 summarises the average values of Young’s modulus of C2S1 and C2S2 adhesive mortars from the tests and calculations using the “Madrid parabola” formulas and the average compressive strengths at thermal actions of 30–50 °C.

The values of Young’s modulus given in Table 6 were supplemented with statistical data—measurement uncertainties of Young’s modulus, which are recorded in Table 7. Since there are two limiting values of Young’s modulus, I treat them as the [min, max] range. The recommended method for an unknown distribution is to assume a rectangular distribution for this range. Standard uncertainty *u* = half-range/$\sqrt{3}$ and expanded uncertainty *U* = 2*u*. The uncertainties in Young’s modulus are very large (e.g., ±1270 MPa) because the range of values (e.g., 5800–8000 MPa) is wide. This strongly suggests that Young’s modulus calculations based on the “Madrid parabola” method at elevated temperatures should not be used.

The analysis of the procedures and calculations described in this article, aimed at verifying the strength parameters of deformable adhesive mortars, aims to support the safe use of C2S1 and C2S2 adhesive mortars, primarily, but not exclusively, in the lightweight underfloor heating system (LRHS). These adhesives can be used in both lightweight and traditional underfloor heating systems, as well as in other applications, typically for bonding ceramic tiles to standard concrete or other substrates, such as wood-like substrates. Importantly, C2S1 and C2S2 adhesive mortars are also exposed to high outdoor temperatures when used to attach ceramic tiles to terraces, stairs, or balconies.

## 4. Conclusions

Experimental testing of the compressive strength of C2S1 and C2S2 deformable adhesive mortars from selected manufacturers under thermal actions enabled the determination of their moduli of elasticity, also known as Young’s moduli, at temperatures of 30 °C, 40 °C, and 50 °C. The experimental study and mathematical calculations verifying the strength parameters of C2S1 and C2S2 adhesives allow us to draw the following conclusions:

1. Based on experimental results, the Young’s modulus and compressive strength of deformable C2S1 adhesive mortars decrease with increasing test temperature. The difference in Young’s modulus between the C2S1 adhesive at 20 °C and 50 °C is approximately 24%.

2. A more accurate picture of the stress–strain relationship resulting in the determination of Young’s modulus, when testing deformable C2S1 and C2S2 adhesive mortars under thermal exposure conditions of 30 °C–50 °C, was obtained by the least squares method, not for a second-degree polynomial, but for a third-degree polynomial.

3. A comparison of the Young’s modulus *E* values obtained from tests and using the so-called “Madrid parabola” formulas shows that calculations based on the “Madrid parabola” significantly overestimate the Young’s modulus values by 23% to 28% for the less deformable adhesive—C2S1—at an exposure temperature of 30–50 °C, and are only slightly higher for the more deformable adhesive C2S2—10% at a test temperature of 30–50 °C. Hence, it is better to base strength calculations using C2S1 and C2S2 adhesive mortars on the Young’s modulus data obtained from the experiment rather than from the “Madrid parabola” calculations.

4. If it is not possible to use experimental testing to obtain Young’s modulus data for deformable cementitious adhesives at elevated temperatures, the calculated Young’s modulus values can be assumed using the “Madrid parabola” formula. However, in this case, the calculated elastic modulus values should be reduced by approximately 25% for C2S1 mortars and by approximately 10% for C2S2 mortars.

5. Based on the experimental studies conducted, the following values of Young’s modulus *E* of deformable cementitious adhesives were assumed to be reliable, depending on the thermal action:

- C2S1; E = 5800 MPa (30 °C), 5600 MPa (40 °C), 4600 MPa (50 °C);
- C2S2; E = 2300 MPa (30 °C–50 °C).

6. The axial compressive strength of C2S1 and C2S2 adhesive mortars decreases with temperature. The difference in compressive strength within the temperature range of 20–50 °C is approximately 50–53% for both deformable adhesive mortars, which should be considered when designing building partitions. The lowest compressive strength value was 6.5 MPa = 6500 kN/m^2^ on average. This does not affect the load-bearing capacity of floors used in residential, public, or industrial buildings where the maximum service loads according to the standard [30] do not exceed 7.5 kN/m^2^.

To verify the Young’s modulus and compressive strength results for Sika C2S1 and Shonox C2S2 deformable adhesive mortars, as well as the proposed percentage reduction in this modulus using the “Madrid parabola” formula, tests should be performed on the same types of cementitious adhesives from other manufacturers.

## Figures and Tables

**Table 1:** Elasticity modulus of concrete (MPa), from [4].

Time	Temperature	0 °C	21 °C	40 °C	121 °C	260 °C
7-day	C20	7319.28	19,762.05	17,036.25	15,236.05	11,474.74
	C27	11,821.12	22,456.88	20,249.47	18,639.21	17,542.78
14-day	C20	3433.34	18,821.00	16,468.38	16,468.38	13,701.69
	C27	7904.82	23,249.47	19,762.05	17,067.23	17,884.21
28-day	C20	3426.62	22,585.20	19,937.14	17,242.92	14,161.26
	C27	12,000.25	23,400.65	20,500.1	17,500.02	18,201.58
56-day	C20	3423.95	26,349.40	19,762.05	17,207.77	15,370.48
	C27	4526.056	34,670.26	24,467.30	18,068.16	19,077.6
90-day	C20	3952.41	27,447.29	22,456.88	20,702.56	18,599.58
	C27	5269.88	35,000.31	29,061.84	23,231.5	21,480.49

**Table 2:** Averaged 3rd degree polynomial functions for C2S1 and C2S2 adhesive mortars.

The Adhesive’s Own Name	A Type of Adhesive That Is Compressed at a Specific Temperature	Averaged 3rd-Degree Polynomial
S1.30	C2S1 Sika Ceram 255 **(30 °C)**	4 × 10^7^x^3^ + 965,502x^2^ + 5813x
S1.40	C2S1 Sika Ceram 255 **(40 °C)**	1 × 10^7^x^3^ + 857,892x^2^ + 5687x
S1.50	C2S1 Sika Ceram 255 **(50 °C)**	4 × 10^7^x^3^ + 841,310x^2^ + 4651x
S2.30	C2S2 Shonox **(30 °C)**	6 × 10^6^x^3^ + 229,098x^2^ + 2468x
S2.40	C2S2 Shonox **(40 °C)**	5 × 10^6^x^3^ + 215,872x^2^ + 2306x
S2.50	C2S2 Shonox **(50 °C)**	8 × 10^6^x^3^ + 259,761x^2^ + 2353x

**Table 3:** Average compressive strength, deformation, displacement, and Young’s modulus at 30 °C, 40 °C and 50 °C of C2S1 and C2S2 adhesive mortars after 6 months of seasoning in the laboratory.

The Adhesive’s Own Name	Adhesive Type and Research Temperature	Calculated and Probabilistic (Approx. Average) Young’s Modulus [MPa]				Compressive Strength/Deformation/Displacement [MPa]/-/mm
		1	2	3	Approx. Average	Average
S1.30	C2S1 Sika Ceram 255 **(30 °C)**	5750	5820	5870	**5800**	**10.1**/0.00377/0.45
S1.40	C2S1 Sika Ceram 255 **(40 °C)**	5704	5594	5652	**5600**	**10.0**/0.00381/0.46
S1.50	C2S1 Sika Ceram 255 **(50 °C)**	4609	4693	4623	**4600**	**8.15**/0.00401/0.49
S2.30	C2S2 Shonox **(30 °C)**	2439	2490	2474	**2400**	**8.44**/0.00933/0.81
S2.40	C2S2 Shonox **(40 °C)**	2129	2398	2390	**2300**	**7.22**/0.00803/0.75
S2.50	C2S2 Shonox **(50 °C)**	2181	2396	2483	**2300**	**6.58**/0.00726/0.73

Test speed: C2S1 and C2S2; V = 0.6 mm/min.

**Table 4:** Measurement uncertainties of the Young’s modulus of C2S1 and C2S2 adhesives.

The Adhesive’s Own Name	Calculated and Probabilistic (Average) Young’s Modulus [MPa]				Expanded Uncertainty *U* (±MPa, k = 2)
	1	2	3	Average	
S1.30	5750	5820	5870	5813	±70
S1.40	5704	5594	5652	5650	±64
S1.50	4609	4693	4623	4642	±52
S2.30	2439	2490	2474	2467	±30
S2.40	2129	2398	2390	2306	±177
S2.50	2181	2396	2483	2353	±180

**Table 5:** The values of Young’s modulus based on tests and calculations and the average compressive strength at 20 °C described in article [23].

Type of Adhesive	Young’s Modulus [MPa]		Compressive Strength [MPa]
	Average Values from Tests	Values from the “Madrid Parabola” Calculation	Average Values from Tests/Calculations
C2S1 Sika Ceram 255 **(20 °C)**	5900	6000	15.5/15.36
C2S2 Shonox **(20 °C)**	1950	2300	13.7/14.26

Test speed: C2S1 and C2S2: V = 0.6 mm/min.

**Table 6:** Average values of Young’s modulus of C2S1 and C2S2 adhesive mortars from tests and calculations using the “Madrid parabola” formulas, as well as average compressive strengths at thermal actions of 30–50 °C.

The Adhesive’s Own Name	Adhesive Type and Research Temperature	Young’s Modulus [MPa]		Compressive Strength [MPa]
		Average Values from Tests	Values from the “Madrid Parabola” Calculation *	Average Values from Tests
S1.30	C2S1 Sika Ceram 255 **(30 °C)**	5800	8000	10
S1.40	C2S1 Sika Ceram 255 **(40 °C)**	5600	7800	9.95
S1.50	C2S1 Sika Ceram 255 **(50 °C)**	4600	6000	7.97
S2.30	C2S2 Shonox **(30 °C)**	2400	2700	8.44
S2.40	C2S2 Shonox **(40 °C)**	2300	2700	7.22
S2.50	C2S2 Shonox **(50 °C)**	2300	2700	6.58

* The values of Young’s modulus from calculations using the “Madrid parabola” formulas were rounded down by analogy to the average values from tests, as well as the recommendations of the article [27].

**Table 7:** Measurement uncertainties of the Young’s modulus of C2S1 and C2S2 adhesives from tests and calculations using the “Madrid parabola” formulas and expanded uncertainty *U* calculated from the half-range assuming a rectangular distribution.

The Adhesive’s Own Name	Young’s Modulus [MPa]		Half-Range [MPa]	Expanded Uncertainty *U* (±MPa, k = 2); Rectangular Distribution
	Average Values from Tests	Values from the “Madrid Parabola” Calculation *		
S1.30	5800	8000	1100	±1270
S1.40	5600	7800	1100	±1270
S1.50	4600	6000	700	±808
S2.30	2400	2700	150	±173
S2.40	2300	2700	200	±231
S2.50	2300	2700	200	±231

* The values of Young’s modulus from calculations using the “Madrid parabola” formulas were rounded down by analogy to the average values from tests, as well as the recommendations of the article [27].

## Data Availability

The original contributions presented in the study are included in the article, further inquiries can be directed to the corresponding author.

## Abbreviations

The following abbreviations are used in this manuscript:

ASTM	American Society for Testing and Materials
C2S1	Cement flexible adhesive
C2S2	Cement highly flexible adhesive
GFRP	Glass Fiber Reinforced Polymer
HPMC	Hydroxy-propylcellulose
LRHS	Lightweight radiant heating system
MTS	Metre-Tonne-Seconde
OSB	Oriented Strand Board
P3AT	poly(3-alkylthiophene)
PCT6BT	poly(9-heptadecanyl)-9H-carbazole-diyl-alt-[7-bis(3-hexylthiophen-5-yl)-2, 3-benzothiadiazole]-2′,2′-diyl
PCDTBT	poly[N-9-heptadecanyl-2,7-carbazole-alt-5,5-(4′,7′-di-2-thienyl-2′,1′,3′-benzothiadiazole)]
PVC	Polyvinyl chloride
RDP	Redispersible polymer powder
XPS	Extruded Polystyrene
